# Inhibition of UBE2N enhances TRAIL-mediated apoptosis through upregulation of DR5 in cancer cells

**DOI:** 10.7150/ijms.130235

**Published:** 2026-07-13

**Authors:** Yu Jin Jeong, Seon Min Woo, Seung Un Seo, So Rae Song, Taeg Kyu Kwon

**Affiliations:** 1 Department of Immunology, School of Medicine, Keimyung University, Daegu 42601, South Korea.; 2 Center for Forensic Pharmaceutical Science, Keimyung University, Daegu 42601, South Korea.

**Keywords:** apoptosis, DR5, NSC697923, TRAIL, UBE2N

## Abstract

Tumor necrosis factor-related apoptosis-induced ligand (TRAIL) selectively induces apoptosis in cancer cells. However, many cancer cells are resistant to TRAIL because of downregulation of death receptors (DRs) and overexpression of anti-apoptotic proteins. Ubiquitin-conjugating enzyme E2N (UBE2N), also known as Ubc13, plays a central role in ubiquitin-mediated cellular activities. In this study, we aimed to explore the sensitization effect of UBE2N inhibition in TRAIL-mediated apoptosis in cancer cells. NSC697923 (a potent inhibitor of UBE2N) alone and TRAIL alone did not induce apoptosis in renal carcinoma Caki cells. However, combined treatment with NSC697923 and TRAIL significantly enhanced apoptotic cell death in cancer cells, but not in normal cells. Mechanistically, NSC697923 induced upregulation of DR5 mRNA and protein levels through CHOP-mediated DR5 transcriptional activation and ubiquitin-mediated DR5 stabilization. NSC697923-mediated DR5 mRNA upregulation was regulated by upregulation of CHOP expression, a key transcriptional factor of DR5. CHOP siRNA treatment inhibited NSC697923-mediated DR5 protein expression. Moreover, NSC697923 generated ROS, and pretreatment with ROS scavengers inhibited DR5 upregulation and NSC697923 plus TRAIL-mediated cell death. These findings suggest that UBE2N inhibitor enhances TRAIL-induced apoptosis by DR5 upregulation and UBE2N inhibition may serve as a potential strategy to overcome TRAIL resistance in cancer therapy.

## Introduction

Tumor necrosis factor-related apoptosis-inducing ligand (TRAIL), a member of the tumor necrosis factor (TNF) superfamily, exhibits cancer-selective cytotoxicity by inducing apoptosis in tumor cells while sparing normal cells 1. TRAIL initiates the extrinsic apoptotic pathway by binding to death receptors (DR4/5), leading to the recruitment of FADD and procaspase-8 and the formation of the death-inducing signaling complex (DISC) 1, 2. TRAIL is known to selectively induce apoptosis in cancer cells while exerting minimal effects on normal cells 3. However, many cancer cells exhibit resistance to TRAIL-mediated apoptosis through various mechanisms, posing a significant hurdle to its clinical application 4. TRAIL resistance is mediated by various mechanisms, including downregulation of DRs expression and overexpression of anti-apoptotic proteins such as the Bcl-2 and IAP subfamily 5-7. To overcome these limitations, several approaches have been developed, including the use of TRAIL receptor agonists, combination therapy, and immunotherapy 8.

Ubiquitination is a type of post-translational modification that involves three enzymes; the E1-activating enzyme, the E2-conjugating enzyme, and the E3 ligase 9. The activated ubiquitin by E1-activating enzyme is transferred to the E2-conjugating enzyme, and E3 ligase is recruited to their complex, eventually increasing the degradation of target substrate by attaching a polyubiquitin chain. Ubiquitin-conjugating enzyme E2 (UBE2), comprising ~40 members in mammals, are implicated in cell cycle, DNA repair, and apoptosis by regulating stabilization and interaction of proteins in cancers 10. UBE2N, also known as Ubc13, mediates K63-linked polyubiquitination and plays a critical role in regulating protein stability and intracellular signaling pathways 11-13. It plays a crucial role in the progression and metastasis of various cancers 14-17. UBE2N promotes tumor development by modulating β-catenin and Wnt signaling pathway, which affects genomic stability in colorectal cancer 16. UBE2N facilitates metastatic colonization and tumor cell survival through activation of the TAK1-p38 MAPK signaling cascade and MEK/FRA1/SOX10 signaling pathway, respectively 17. Inhibition of UBE2N by NSC697923 induces apoptosis via activation of the p53 and JNK pathways in neuroblastoma 18. More recent studies have implicated UBE2N in the regulation of tumor survival and drug resistance in ovarian cancer, prostate cancer, and acute myeloid leukemia 19-21.

In this study, we aimed to elucidate a novel molecular mechanism by which UBE2N modulates DR5 expression, thereby enhancing TRAIL sensitivity in cancer cells. This work may provide a new therapeutic strategy to overcome the limitations associated with TRAIL-based therapies.

## Materials and Methods

### Cells and materials

Human renal carcinoma (Caki and ACHN), colon cancer (HCT116), prostate cancer (PC3) cells were obtained from the American Type Culture Collection (Manassas, VA, USA). Human normal vascular endothelial cells EA.hy926 cells were gifted by Dr. T.J. Lee (Yeungnam University, Daegu, South Korea). The cells were cultured in the Dulbecco's modified Eagle's medium containing 10% fetal bovine serum (Cytiva, Logan, UT, USA), 1% penicillin/streptomycin (Thermo Scientific, Waltham, MA, USA), and 100 g/mL gentamicin (Thermo Scientific) 22. TRAIL and z-VAD-fmk were purchased from R&D Systems (Minneapolis, MN, USA). Other chemicals were obtained from Sigma-Aldrich (St. Louis, MO, USA). The information on primary antibodies was provided as below: anti-DR5, anti-cIAP1, anti-Mcl-1, anti-Bcl-xL, anti-Bax, anti-CHOP, anti-PARP and anti-cleaved caspase-3 from Cell Signaling Technology (Beverly, MA, USA); anti-UBE2N, anti-cIAP2, anti-Bcl-2 and anti-p53 from Santa Cruz Biotechnology (Santa Cruz, CA, USA); anti-Survivin from R&D system (Minneapolis, MN, USA); anti-caspase-3 and anti- c-FLIP from Enzo Life Sciences (Ann Arbor, MI, USA); anti-XIAP from BD Biosciences (San Jose, CA, USA); anti-DR4 and anti-Bim from Abcam (Cambridge, England); anti-actin from Sigma-Aldrich.

### Flow cytometry analysis

For cell cycle analysis, cells were harvested and resuspended in 100 μL of phosphate-buffered saline (PBS), followed by the addition of 200 μL of 95% ethanol while vortexing. The cells were incubated at 4°C for 1 h. The cell pellet was resuspended in 200 μL of 1.12% sodium citrate buffer (pH 8.4) containing RNase A and incubated at 37°C for 30 minutes. And then, propidium iodide was added for DNA staining. The stained cells were analyzed by flow cytometry (BD Biosciences) to determine the sub-G1 population.

### Western blotting

Cells were lysed using ERK lysis buffer, and equal amounts of protein were separated by sodium dodecyl sulfate-polyacrylamide gel electrophoresis (SDS-PAGE) and transferred to a nitrocellulose membrane (GE Healthcare Life Science, Pittsburgh, PA, USA). The membranes were incubated overnight at 4°C with primary antibodies, followed by incubation with HRP-conjugated secondary antibodies for 2 hours at room temperature. Protein signals were detected using an enhanced chemiluminescence (ECL) kit (Merck Millipore, Darmstadt, Germany).

### Measurement of DNA fragmentation

To assess nuclear condensation, cells were stained with 300 nM 4′,6-diamidino-2-phenylindole (DAPI; Roche, Mannheim, Germany) for 5 minutes and observed using a fluorescence microscope (Carl Zeiss, Jena, Germany). DNA fragmentation was evaluated using a Cell Death Detection ELISA Plus Kit (Boehringer Mannheim, Indianapolis, IN, USA).

### Small interfering RNA (siRNA)

The cells were transfected with the control, UBE2N, and DR5 siRNA using Lipofectamine RNAiMAX (Thermo Fisher Scientific). The siRNAs were obtained from Santa Cruz Biotechnology.

### Quantitative real-time PCR (qPCR)

Total RNA isolation, complementary DNA (cDNA) synthesis, and qPCR were performed as previously described 23. mRNA amplification was assessed using the Thermal Cycler Dice® Real-Time System III (Takara Bio Inc., Shiga, Japan) and determined using the 2^-ΔΔCt^ method. The primers used for DR5 and actin amplification were as follows: DR5 (forward) 5′-AGA CCC TTG TGC TCG TTG TC -3′, (reverse) 5′-TTG TTG GGT GAT CAG AGC AG -3′; actin (forward) 5′-CTA CAA TGA GCT GCG TGT G-3′, (reverse) 5′-TGG GGT GTT GAA GGT CTC -3′*.*

### Measurement of DR5 expression on cell surface

For cell surface expression analysis of DR5, the cells were stained using DR5-phycoerythrin (Abcam) in PBS containing 10% FCS and 1% sodium azide, and then analyzed using flow cytometry (BD Biosciences) 24.

### Ubiquitination assay

Cells were collected and washed with PBS containing 10 mM N-ethylmaleimide (NEM), then resuspended in 90 μL of PBS/NEM containing 1% SDS and heated at 95°C for 10 minutes. The lysates were diluted with buffer containing 1 mM phenylmethylsulfonyl fluoride (PMSF) and 5 mM NEM, and sheared using a 1 mL syringe. After centrifugation at 13,000 × g for 10 minutes at 4°C, the supernatants were incubated overnight with primary antibodies against the target protein. Protein-antibody complexes were incubated with agarose beads for 2 hours, followed by washing twice with buffer containing 1 mM PMSF and 5 mM NEM. Bound proteins were eluted by boiling in 2× sample buffer for 10 minutes. Ubiquitinated DR5 was detected using an HRP-conjugated anti-ubiquitin antibody.

### Measurement of ROS generation

Intracellular ROS generation was analyzed using 20 μM H_2_DCF-DA (Thermo Fisher Scientific). Before harvesting, the cells were stained with H_2_DCF-DA dye for 10 min, following which the cells were trypsinized and resuspended in PBS, and ROS production was measured using a BD Accuri^TM^ C6 cytometer (BD Biosciences).

### Statistical analysis

All the statistical analyses were performed using the Statistical Package for Social Sciences (SPSS, version 26.0; IBM Corp., Armonk, NY, USA). All experiments were performed in triplicates. Data are presented as means ±standard deviations (SDs) and were analyzed using one-way analysis of variance (ANOVA) and post hoc comparisons (Student- Newman-Keuls test).

## Results

### Inhibition of UBE2N sensitizes Caki cells to TRAIL-induced apoptosis

**Figure 1:**
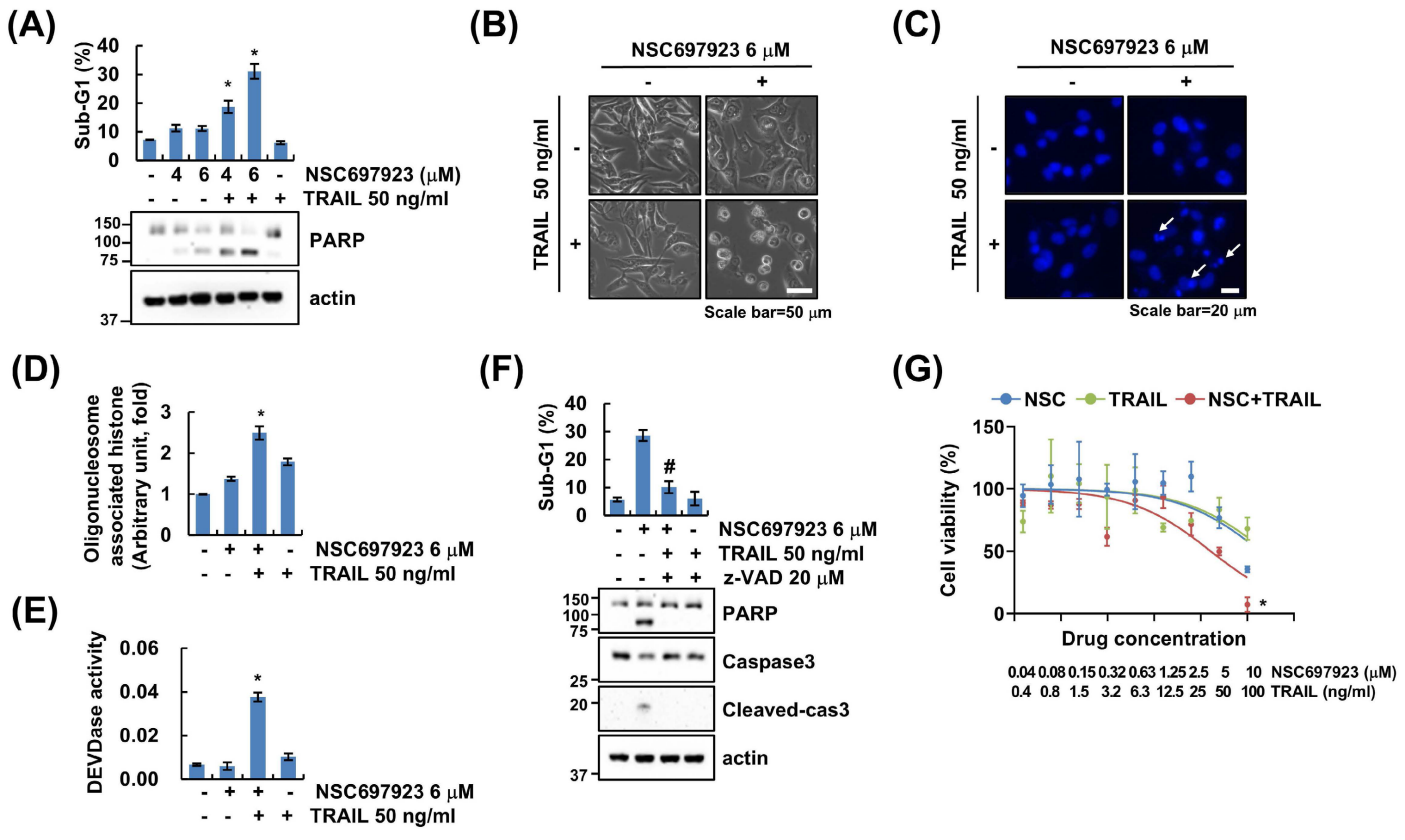
** NSC697923 sensitizes Caki cells to TRAIL-induced apoptosis. (A)** Caki cells were treated with 4, 6 μM NSC697923 and/or 50 ng/ml TRAIL for 24 h. **(B-E)** Caki cells were treated with 6 μM NSC697923 and/or 50 ng/ml TRAIL for 24 h. Cell morphology was examined using interference light microscopy **(B)**. Condensation and fragmentation of nuclei were examined by DAPI staining **(C)** and DNA fragmentation kit **(D)**, respectively. Caspase-3 activity was detected using DEVDase substrate **(E)**. **(F)** Caki cells were pretreated with 20 μM z-VAD for 30 min, followed by treated with 6 μM NSC697923 and 50 ng/ml TRAIL for 24 h. **(G)** Drug dose-response curves for Caki cells to combination of NSC697923 and TRAIL. The sub-G1 population and protein expression were measured by flow cytometry (A, D, E and F) and western blotting (A and F). *p<0.05 compared to control. #*p*<0.05 compared to combination treatment with NSC697923 and TRAIL**.**

To investigate whether UBE2N inhibition influences sensitivity to TRAIL-induced apoptosis, Caki (renal cell carcinoma) cells were treated with either a UBE2N inhibitor (NSC697923, 4 or 6 μM), TRAIL (50 ng/mL), or a combination of both agents. Neither monotherapy induced apoptosis; however, combined treatment resulted in a dose-dependent increase in the sub-G1 population (Figure 1A). Apoptotic morphological changes, including cell shrinkage and reduced cell volume, were observed following combination treatment (Figure 1B). Cotreatment with NSC697923 and TRAIL increased nuclear condensation and DNA fragmentation, characteristics of apoptosis (Figure 1C and D). Moreover, caspase-3 (DEVDase) activity was increased upon co-treatment (Figure 1E), and pan-caspase inhibitor z-VAD-fmk completely abrogated apoptosis by NSC697923 and TRAIL co-treatment (Figure 1F). To confirm the sensitizing effect of NSC697923 on TRAIL-induced apoptosis, we performed dose-response analyses. Co-treatment with NSC697923 and TRAIL significantly decreased cell viability in a concentration-dependent manner (Figure 1G). These findings suggest that inhibition of UBE2N markedly enhances TRAIL-mediated apoptosis in Caki cells.

### Knockdown of UBE2N upregulates DR5 expression

**Figure 2:**
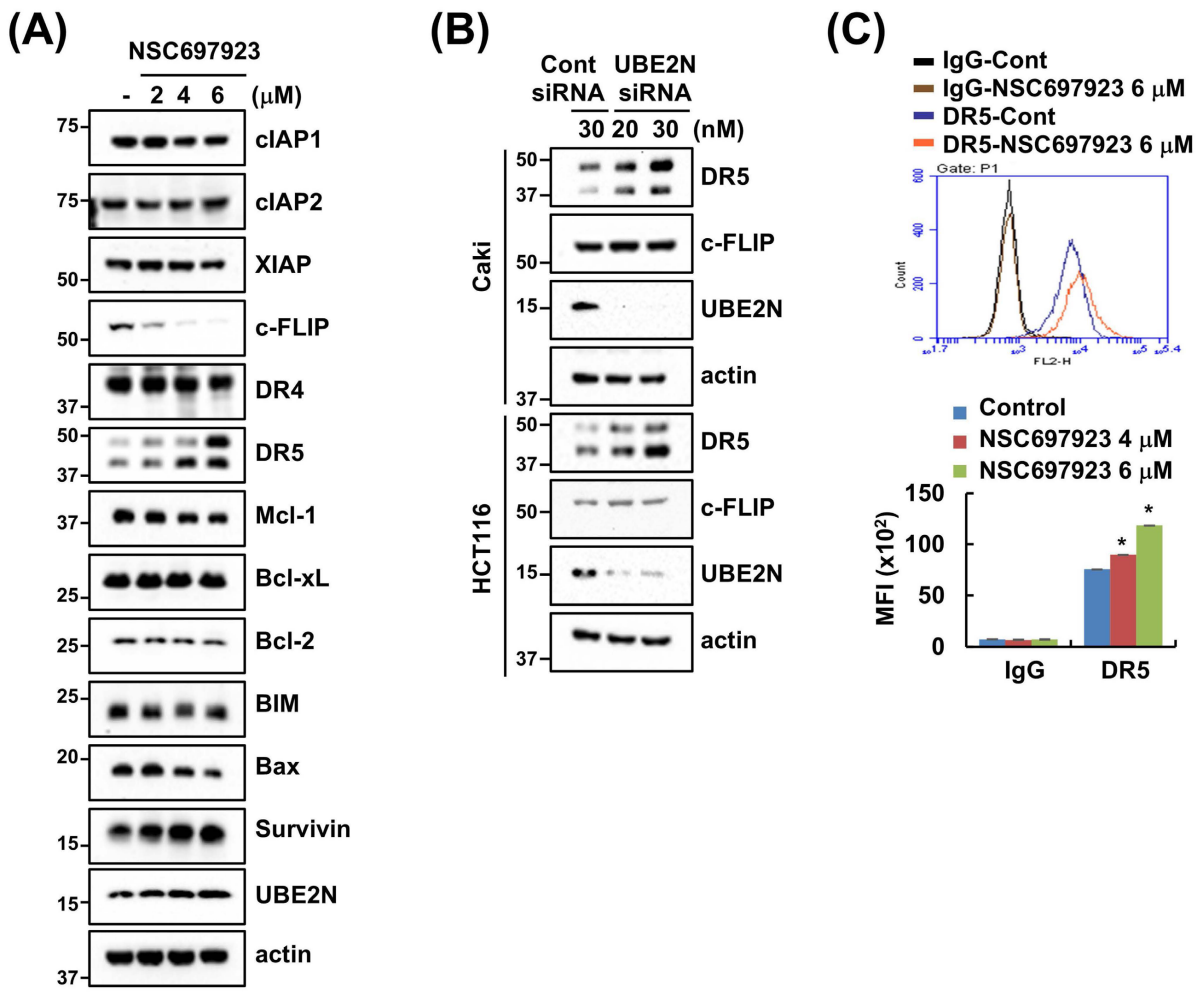
** Inhibition of UBE2N upregulates DR5 expression. (A)** Caki cells were treated with 2-6 μM NSC697923 for 24 h. **(B)** Caki and HCT116 cells were transfected with control and UBE2N siRNA for 24 h. **(C)** Caki cells were treated with 2-6 μM NSC697923 for 24 h. DR5 expression on surface was detected using flow cytometry. Protein expression was measured by western blotting (A and B). *p<0.05 compared to control.

Next, we assessed the effect of NSC697923 on the regulation of apoptosis-related proteins. DR5 expression was upregulated, whereas c-FLIP expression was downregulated by NSC697923 (Figure 2A). However, the expression levels of other apoptosis-related proteins are unaffected. Moreover, since NSC697923 inhibits the enzyme activity of UBE2N by covalently binding at the active site (cysteine 87) of UBE2N 25, NSC67923 did not alter UBE2N protein expression (Figure 2A). To further validate the functional role of UBE2N, Caki and HCT116 cells were transfected with UBE2N-specific siRNA. Knockdown of UBE2N using siRNA resulted in the upregulation of DR5 expression; however, c-FLIP expression did not alter (Figure 2B). In addition, NSC697923 increased DR5 expression level on surface (Figure 2C). Therefore, since NSC697923-mediated c-FLIP downregulation was off-target effect, we focused on elucidating underlying the molecular mechanism of DR5 regulation by UBE2N inhibition.

### Inhibition of UBE2N stabilizes DR5 protein expression and inhibits its ubiquitination

**Figure 3:**
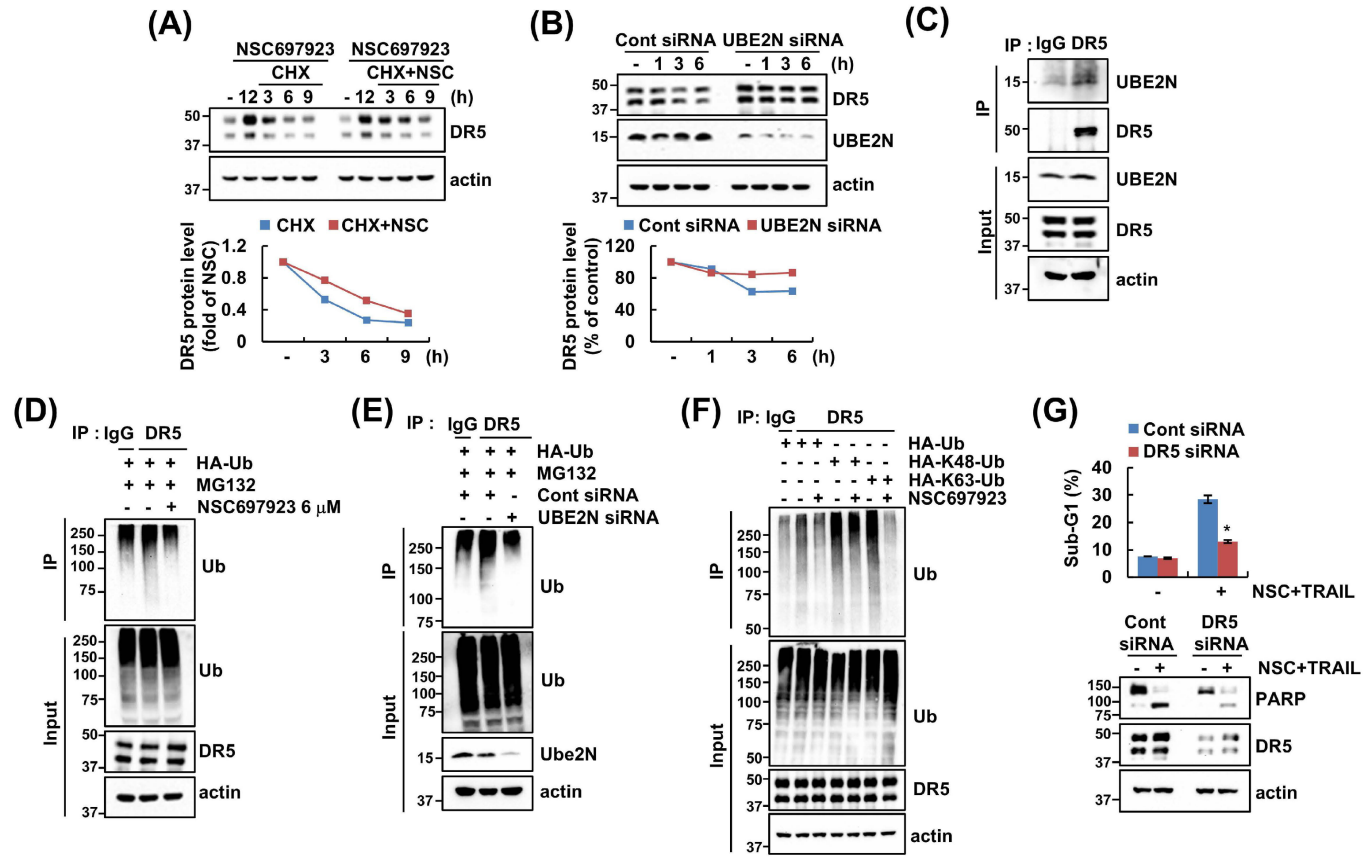
** NSC697923 stabilizes DR5 protein expression and inhibits its ubiquitination. (A)** Caki cells were treated with 6 μM NSC697923 for 12 h, and then treated with 20 μg/mL CHX with or without 6 μM NSC697923 for the indicated time periods. **(B)** Caki cells were transfected with control and UBE2N siRNA and then treated 20 μg/mL CHX for the indicated time periods. **(C)** The interaction of UBE2N and DR5 was demonstrated by immunoprecipitation (IP). (D) Caki cells were transfected with HA-ubiquitin (HA-Ub) plasmid for 24 h. Then cells were treated with 0.5 μM MG132 and 6 μM NSC697923 for 24 h. **(E)** Caki cells were co-transfected control and UBE2N siRNA with HA-Ub plasmid. DR5 ubiquitination was detected by western blotting using HRP-conjugated anti-Ub antibody. **(F)** Caki cells were transfected with HA-Ub, HA-K48-Ub, or HA-K63-Ub plasmid for 24 h. Then cells were treated with 0.5 μM MG132 and 6 μM NSC697923 for 24 h. **(G)** Caki cells were transfected control siRNA or DR5 siRNA, and then treated with combinations treatment of NSC697923 and 50 ng/mL TRAIL for 24 h. The sub-G1 population and protein expression were measured by flow cytometry (F) and western blotting (A-F). The band intensity was expected using Image J (A and B). *p<0.05 compared to combination treatment with NSC697923 and TRAIL in control siRNA.

To investigate the post-translational regulation of DR5 expression upon inhibition of UBE2N, we examined the DR5 protein stability using cycloheximide (CHX), a protein synthesis inhibitor. CHX alone degraded DR5 protein level sustained by NSC697923 from 6 h, whereas cotreatment with CHX and NSC697923 retained DR5 protein expression to 9 h (Figure 3A). Furthermore, deletion of UBE2N also resulted in prevention of DR5 degradation compared to control siRNA, indicating the UBE2N-mediated DR5 stabilization in at the post-translational level (Figure 3B).

To determine whether UBE2N directly interacts with DR5, immunoprecipitation (IP) assays were performed. DR5 interacts with UBE2N, suggesting that UBE2N may directly regulate DR5 protein stability (Figure 3C). Given that UBE2N is an E2 enzyme involved in ubiquitination, we investigated whether UBE2N inhibition affects the ubiquitination of DR5. Inhibitor and knockdown of UBE2N reduced the polyubiquitination of DR5, suggesting that UBE2N may facilitate DR5 degradation by promoting its ubiquitination (Figure 3D and E). Since UBE2N is known to primarily mediate K63-linked polyubiquitination, we performed ubiquitination assay using linkage-specific ubiquitin plasmid. NSC697923 markedly decreased K63-linked ubiquitination of DR5, whereas K48-linked ubiquitination remained unaffected (Figure 3F). These data suggest that UBE2N regulates DR5 stability through K63-linked ubiquitination rather than typical K48-linked proteasomal degradation. Moreover, depletion of DR5 significantly suppressed the combinations of NSC697923 and TRAIL-mediated apoptosis (Figure 3G). Collectively, these results suggest that UBE2N interacts with and ubiquitinates DR5 protein, thereby increasing TRAIL sensitivity through inhibition of UBE2N-mediated DR5 upregulation.

### NSC697923 increases DR5 mRNA expression through CHOP-mediated transcriptional activation

**Figure 4:**
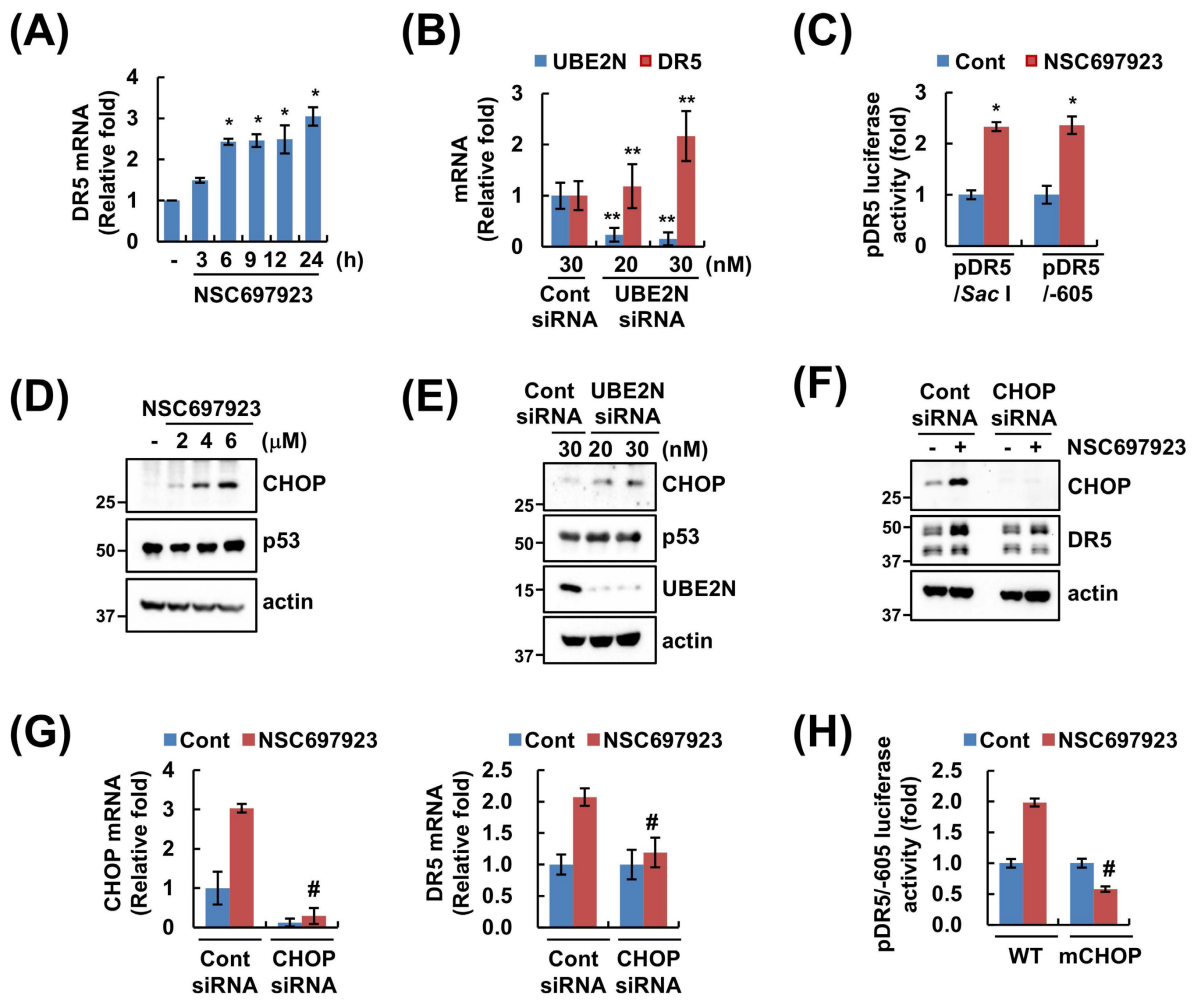
** NSC697923 increases DR5 mRNA expression through CHOP-mediated transcriptional activation. (A)** Caki cells were treated with 6 μM NSC697923 for the indicated time periods. DR5 mRNA expression was measured by qRT-PCR. **(B)** Caki cells were transfected with control and UBE2N siRNA for 24 h. The mRNA expression was measured by qRT-PCR. **(C)** Caki cells were transfected with pDR5/*Sac* I or pDR5/-605 promoter plasmid and treated with 6 μM NSC697923 for 12 h. After treatment, the cells were lysed, and the luciferase activity was analyzed. **(D)** Caki cells were treated with 6 μM NSC697923 for 9 h. **(E)** Caki cells were transfected with control and UBE2N siRNA for 24 h. **(F-G)** Caki cells were transfected with control and CHOP siRNA and treated with 6 μM NSC697923 for 9 h. **(H)** Caki cells were transfected with pDR5/-605 WT or pDR5/-605 CHOP mutant (mCHOP) promoter plasmid and treated with 6 μM NSC697923 for 12 h. After treatment, the cells were lysed, and the luciferase activity was analyzed. Protein (D-F) and mRNA (A, B, and G) expression were measured by western blotting and qRT-PCR, respectively. *p<0.05 compared to control. **p<0.05 compared to control siRNA. #*p*<0.05 compared to NSC697923 in control siRNA or pDR5/-605.

Interestingly, NSC697923 and UBE2N knockdown induced upregulation of DR5 mRNA expression (Figure 4A and B). To further confirm the transcriptional regulation of DR5 after NSC697923 treatment, luciferase reporter assay was performed. Two different promoter plasmids were employed: DR5/SacI, which contains an extended promoter region up to the SacI site, and DR5/-605, which includes a shorter region up to -605 bp. NSC697923 significantly enhanced luciferase activity driven by both promoter constructs (Figure 4C). Previous studies have identified CHOP and p53 as transcription factors involved in the regulation of the DR5 promoter 26, 27. Inhibitor and siRNA of UBE2N upregulated CHOP expression, whereas p53 levels remained unchanged (Figure 4D and E). To determine the functional relevance of CHOP, siRNA-mediated knockdown of CHOP was performed. Notably, ablation of CHOP significantly attenuated the NSC697923-induced upregulation of DR5 protein and mRNA level (Figure 4F and G). Consistent with this, mutant DR5 promoter construct lacking the CHOP-binding site demonstrated that NSC697923 is unable to enhance luciferase activity (Figure 4H). Taken together, these data indicate that NSC697923 increases CHOP expression, thereby contributing to upregulation of DR5 mRNA expression.

### NSC697923 induces ROS generation, which promotes DR5 expression and TRAIL sensitivity

**Figure 5:**
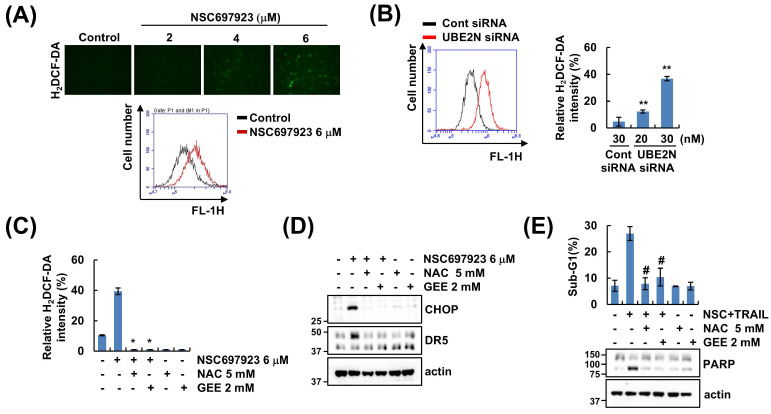
** NSC697923-increased ROS generation is involved in TRAIL sensitivity. (A)** Caki cells were treated with 2-6 μM NSC697923 for 30 min and then the cells were loaded with H_2_DCF-DA fluorescent dye. **(B)** Caki cells were transfected with control and UBE2N siRNA and then the cells were loaded with H_2_DCF-DA fluorescent dye. **(C-D)** Caki cells were pretreated with 5 mM NAC or 2 mM GEE for 30 min, and then treated with 6 μM NSC697923 for 30 min. The cells were loaded with H_2_DCF-DA fluorescent dye **(C)**. Protein expression was measured by western blotting **(D)**.** (E)** Caki cells were pretreated with 5 mM NAC or 2 mM GEE for 30 min, and then treated with combinations of 6 μM NSC697923 and 50 ng/ml TRAIL for 24 h. The sub-G1 population and protein expression were measured by flow cytometry and western blotting. *p<0.05 compared to NSC697923. **p<0.05 compared to control siRNA. #*p*<0.05 compared to combination treatment with NSC697923 and TRAIL.

Given that intracellular reactive oxygen species (ROS) is associated with TRAIL sensitization, we explored the effect of a UBE2N inhibitor on ROS generation. Fluorescence imaging and flow cytometry analysis demonstrated that NSC697923 markedly elevated intracellular ROS levels (Figure 5A). To determine whether UBE2N inhibition induces ROS generation, we measured ROS level following UBE2N knockdown. Depletion of UBE2N increased ROS levels (Figure 5B). ROS scavengers [N-acetylcysteine (NAC) or GEE] completely blocked NSC697923-induced ROS generation (Figure 5C). Next, we investigated whether NSC697923-increased ROS level regulates DR5 expression and the sensitivity to TRAIL. Western blot was performed to check the alteration of NSC697923-mediated CHOP and DR5 upregulation upon antioxidants, and these results showed that pretreatment of antioxidants significantly inhibits their expression induced by NSC697923 (Figure 5D). Furthermore, combined treatment with NSC697923 and TRAIL-induced apoptosis was suppressed by ROS scavenger (Figure 5E). These findings demonstrate that ROS generation plays an essential role in the CHOP and DR5 upregulation and TRAIL sensitization upon UBE2N inhibition.

### Effect of UBE2N inhibition on TRAIL-induced apoptosis in normal cell lines

**Figure 6:**
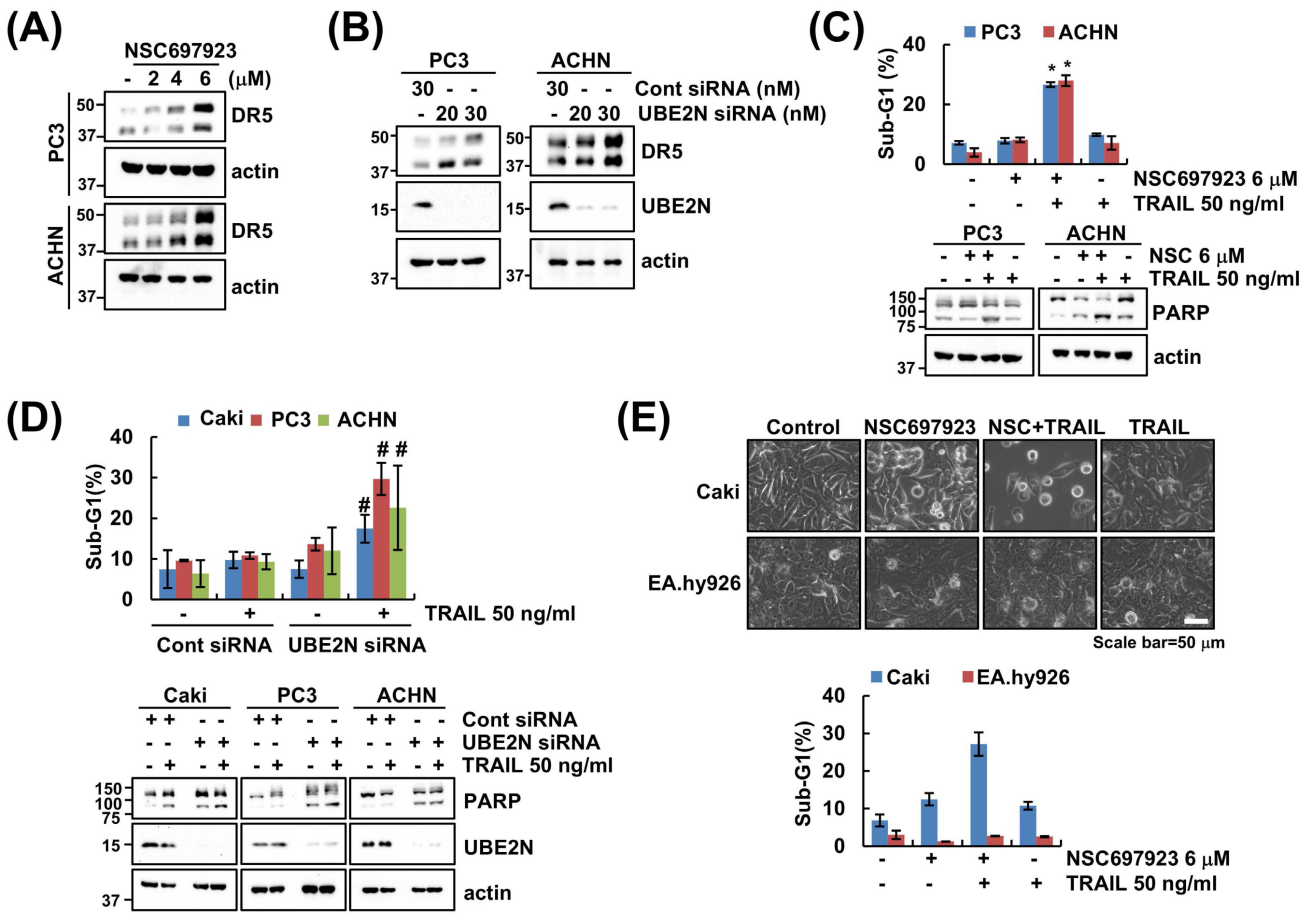
** Inhibition of UBE2N increases TRAIL sensitivity in multiple cancer cell lines but not in normal endothelial cells. (A)** PC3 and ACHN cells were treated with the indicated concentrations of NSC697923 for 24 h. **(B)** PC3 and ACHN cells were transferred control siRNA or UBE2N siRNA for 24 h. **(C)** PC3 and ACHN were treated with 6 μM NSC697923 or/and 50 ng/ml TRAIL for 24 h. **(D)** Caki, PC3 and ACHN cells were transfected control siRNA or UBE2N siRNA and then treated with 50 ng/mL TRAIL for 24 h. **(E)** Normal endothelial EA.hy926 cells were treated with 6 μM NSC697923 or/and 50 ng/ml TRAIL for 24 h. The sub-G1 population and protein expression were measured by flow cytometry (C-E) and western blotting (A-D). *p<0.05 compared to control. #*p*<0.05 compared to TRAIL in control siRNA.

We next sought to determine the effect of UBE2N inhibition in other cancer cell types. Treatment with the NSC697923 led to a marked increase in DR5 expression in both prostate cancer cells (PC3) and the renal cancer cells (ACHN) cells (Figure 6A). Consistently, knockdown of UBE2N via siRNA also elevated DR5 levels in both cell lines (Figure 6B). Furthermore, pharmacological (NSC697923) and genetic (siRNA) inhibition of UBE2N significantly enhanced TRAIL-mediated apoptosis, as evidenced by an increase in the sub-G1 population and PARP cleavage (Figure 6C and D). Finally, to assess the cancer cell specificity of this mechanism, we examined the apoptotic effect of combination therapy in human normal vascular endothelial cells (EA.hy926). Expectably, NSC697923 and TRAIL co-treatment did not induce significant apoptosis in EA.hy926 cells (Figure 6E), suggesting that inhibition of UBE2N selectively enhances TRAIL sensitivity in cancer cells while sparing normal cells.

## Discussion

This study identified a novel mechanism by which the ablation of UBE2N enhances TRAIL sensitivity in cancer cells through the upregulation of DR5 expression. NSC697923 reduced DR5 ubiquitination, thus increasing its protein stability. In addition, NSC697923 promoted CHOP-dependent DR5 mRNA expression at the transcriptional regulation. These effects were consistently observed across multiple cancer cell lines, while no significant apoptosis was induced in normal cells, indicating the potential for a tumor-selective therapeutic strategy. Our findings suggest that targeting the UBE2N-DR5 axis may offer a promising approach for cancer therapy.

Interestingly, we also observed a reduction in DR5 polyubiquitination and induction in DR5 mRNA expression following UBE2N depletion, indicating that UBE2N can modulate DR5 expression through both post-translational and transcriptional regulatory mechanisms. First, inhibition of UBE2N (NSC697923 and siRNA) significantly suppressed DR5 ubiquitination, thereby enhancing DR5 protein stabilization (Figure 3A, B, D, and E). Furthermore, NSC697923 decreased K63-linked ubiquitination of DR5 without affecting K48-linked ubiquitination (Figure 3F), suggesting that UBE2N inhibition maintains DR5 protein stability through non-canonical mechanisms involving the inhibition of K63-linked ubiquitination. Typically, K63-linked ubiquitination is involved in non-proteasomal degradation processes, such as endocytic trafficking or lysosomal degradation; thus, our data suggest that UBE2N-mediated K63-linked ubiquitination plays a role in determining the subcellular localization or protein turnover of DR5. Although the crucial trafficking pathways remain to be further elucidated, these results identify UBE2N as a key regulator of DR5 turnover via a non-canonical mechanism. Previous study reported that E3 ligase c-Cbl is involved in DR5 degradation and TRAIL resistance 28. To investigate whether c-Cbl mediates the DR5 accumulation by UBE2N inhibition, we utilized a c-Cbl overexpression system. Notably, overexpression of c-Cbl failed to abrogate NSC697923-mediated DR5 upregulation (data not shown). Although our findings clearly suggest a UBE2N-dependent DR5 destabilization, the specific E3 ligase partnering with UBE2N for DR5 ubiquitination remains to be elucidated.

Multiple transcription factors such as p53, CHOP, NF-κB, Sp1, FOXO, and YY1 were involved in the transcriptional regulation of DR5 29, 30. We found that NSC697923 induces CHOP expression, but not p53 (Figure 4D). Therefore, we investigated the involvement of CHOP on transcriptional regulation of DR5 by NSC697923. NSC697923 increased luciferase activity of DR5 WT, whereas CHOP-mutated DR5 luciferase activity was not altered (Figure 4H). Moreover, CHOP knockdown markedly suppressed NSC697923-increased expression of DR5 mRNA (Figure 4G). These findings suggest that UBE2N inhibition-induced CHOP expression has a critical role in upregulation of DR5 mRNA. Furthermore, since CHOP is a well-known ER stress marker, we investigated the involvement of ER stress pathway in NSC697923-mediated DR5 upregulation and TRAIL sensitization using chemical ER inhibitors (TUDCA and 4-PBA). Unexpectedly, these inhibitors did not prevent the DR5 accumulation and TRAIL sensitivity by NSC697923 treatment (data not shown). Therefore, these results suggest that UBE2N-DR5 axis plays a major role in TRAIL sensitization, rather than the induction of ER stress. Furthermore, we examined the induction of DNA damage in NSC697923-mediated TRAIL sensitization, as UBE2N inhibition is known to affect DNA repair pathway 25, 31. However, NSC697923 alone slightly increased γH2AX expression, whereas the combination of NSC697923 and TRAIL led to a robust increase (data not shown). These data suggest that DNA damage is a downstream consequence of the apoptotic process rather than upstream of DR5 upregulation.

The generation of ROS has been demonstrated to play a critical role in the observed DR5 upregulation and enhanced TRAIL sensitivity 32, 33. Genetic (siRNA) and pharmacological (NSC697923) inhibition of UBE2N significantly increased intracellular ROS level (Figure 5A and B). However, ROS scavengers prevented CHOP-dependent DR5 upregulation and TRAIL sensitization by NSC697923 (Figure 5D and E). Therefore, these data demonstrate that ROS acts as an upstream mediator of CHOP activation and DR5 upregulation, providing a robust hierarchical link to TRAIL sensitization. To further examine the source of ROS increased by UBE2N inhibition, we utilized various organelle-specific inhibitors, including a mitochondrial ROS inhibitor (MitoTEMPO) and NADPH oxidase (NOX) inhibitors (diphenyleneiodonium and apocynin). However, these inhibitors failed to decrease combinations of NSC697923 and TRAIL-induced apoptosis (data did not shown). These findings suggest that ROS generation may not be predominantly derived from these sources, but rather reflect cellular stress responses induced by UBE2N inhibition. Further studies will be required to elucidate the molecular mechanisms linking UBE2N inhibition to ROS generation, particularly in the context of cellular stress responses.

From a clinical perspective, TRAIL-based cancer therapy offers the advantage of inducing tumor-specific apoptosis, yet its efficacy is limited by the intrinsic resistance observed in many cancer types 34, 35. Our study demonstrated that inhibition of UBE2N increases DR5 expression and sensitizes cancer cells to TRAIL, highlighting its potential as a combinatorial therapeutic strategy to overcome TRAIL resistance. Consistent with this therapeutic potential, co-treatment with a UBE2N inhibitor and TRAIL did not induce significant apoptosis in normal human endothelial cells (Figure 6E), indicating tumor-selective cytotoxicity. Furthermore, previous reports have demonstrated that NSC697923 reduces tumor growth in many xenograft mouse models without any detectable toxicity 18, 36. Taken together, these in vivo findings and our observations in normal cells provide a rationale for developing NSC697923 as a TRAIL sensitizer in clinical applications.

In summary, this study proposes a novel mechanism by which UBE2N inhibition upregulates DR5 expression and enhances TRAIL sensitivity, suggesting that DR5 may represent a previously unrecognized substrate of UBE2N.

## Data Availability

The data used in this study were available from the corresponding author upon reasonable request.
